# The role of inflammatory miRNA–mRNA interactions in PBMCs of colorectal cancer and obesity patients

**DOI:** 10.1002/iid3.702

**Published:** 2022-10-27

**Authors:** Morteza Gholami, Marziyeh Zoughi, Bagher Larijani, Rasoul Abdollahzadeh, Reza Taslimi, Zeinab Rahmani, Alireza Kazemeini, Roobic Behboo, Farideh Razi, Milad Bastami, Shirin Hasani‐Ranjbar, Mahsa M. Amoli

**Affiliations:** ^1^ Metabolic Disorders Research Center, Endocrinology and Metabolism Molecular‐Cellular Sciences Institute Tehran University of Medical Sciences Tehran Iran; ^2^ Obesity and Eating Habits Research Center, Endocrinology and Metabolism Clinical Sciences Institute Tehran University of Medical Sciences Tehran Iran; ^3^ Endocrinology and Metabolism Research Center, Endocrinology and Metabolism Clinical Sciences Institute Tehran University of Medical Sciences Tehran Iran; ^4^ Department of Medical Genetics, School of Medicine Tehran University of Medical Sciences Tehran Iran; ^5^ Department of Gastroenterology, Imam Khomeini Hospital Tehran University of Medical Sciences Tehran Iran; ^6^ Department of General Surgery, Imam Khomeini Hospital, School of Medicine Tehran University of Medical Sciences Tehran Iran; ^7^ Hazrate Rasoole Akram Hospital Iran University of Medical Science Tehran Iran; ^8^ Metabolomics and Genomics Research Center, Endocrinology and Metabolism Molecular‐Cellular sciences institute Tehran University of Medical Sciences Tehran Iran; ^9^ Department of Medical Genetics, Faculty of Medicine Tabriz University of Medical Sciences Tabriz Iran

**Keywords:** colorectal neoplasms, genes, inflammation, microRNAs, obesity

## Abstract

**Introduction:**

Inflammation is a critical hallmark in obesity and colorectal cancer (CRC). This study aimed to investigate effective microRNA (miRNA)–messenger RNA (mRNA) interactions on inflammatory networks involved in obesity and CRC.

**Methods:**

The literature searches were applied to identify genes expression reported on peripheral blood mononuclear cells (PBMCs) and/or blood of CRC subjects and to find inflammatory miRNA  in blood samples. Furthermore, bioinformatics analysis was utilized to find inflammatory miRNA:mRNA interactions of the genes. Finally, a case‐control study was set to investigate the expression of *LAMC1* and *GNB3* genes besides miR‐10b, miR‐506‐3p, miR‐150‐5p, and miR‐124‐3p in CRC and control subjects.

**Results:**

The expression of *LAMC1* gene in healthy control groups was associated with body mass index (BMI) (*p* < .05). The level of miR‐10b (*p <* .001), miR‐506 (*p <* .001), and miR‐124 (*p <*. 001) were significantly increased in PBMCs of CRC patients, while they were not associated with BMI. The level of miR‐150 was associated with BMI in healthy subjects (*p <* .05).

**Conclusions:**

The changes in the level of miR‐506 and miR‐124 in CRC patients may be associated with the regulatory role of these miRNAs on *LAMC1* expression. The *LAMC1* may be related to BMI, however, more observational studies on other populations are needed.

## Graphical abstract

This study aimed to investigate effective miRNA–mRNA interactions on inflammatory networks involved incolorectal cancer (CRC) and obesity. For the first time, we combined literature reviews, bioinformatics, and case‐control studies, to find new inflammatory markers (miRNAs and genes) associated with colorectal cancer and obesity. The results showed that miR‐506‐3p:*LAMC1* and miR‐124‐3P:*LAMC1* interactions are associated with CRC, the expression of miR‐10b‐5p is increased in peripheral blood mononuclear cells of CRC patients, and the expression of miR‐150‐5p and *LAMC1* are associated with body mass index.

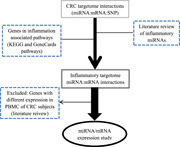

## 1 INTRODUCTION

Cancers are the most common causes of death after cardiovascular diseases. About 30%–50% of cancer can be prevented by managing their risk factors.^1^ The relationship between obesity and some cancers has been identified in previous studies.^2^ Obesity is confirmed as a risk factor in common types of cancers such as colorectal cancer (CRC).^2^, ^3^ According to global cancer statistics in 2018, about 6.1% of cancer incidence and 9.2% of deaths are related to CRC.^4^ Identifying new noninvasive markers for early diagnosis and risk of CRC disease is essential for their treatment. Obesity is associated with an increased risk of CRC.^5^ Accordingly, evidence emphasizes the prevention of malignancies by avoiding obesity^6^ and as the prevalence of obesity increases, precise strategies are needed to identify the link between this disease and development of various types of cancers.^7^

Obesity is a chronic inflammatory disease and inflammation is associated with cancer and can play an effective role in cancer development.^8^ The immune parameters are important regulators in cancer development.^9^ Cancer and inflammation use similar mechanisms of development.^10^ Epidemiological studies have shown that up to 25% of cancers are associated with chronic inflammation.^11^ Several studies have investigated the role of inflammation in CRC,^12^ obesity^13^ Obesity‐related inflammatory responses in adipose tissue may play an important role in tumorigenesis.^14^ Inflammatory responses in cells release mediators that will increase cell proliferation, stimulate angiogenesis, and prevent cell death.^14^ Approximately 1/4 of cancers are associated with chronic infections and inflammation.^11^ Inflammatory pathways such as IκBα kinase β (IKKβ) and Jun *N*‐terminal kinase (JNK1) are activated in obese people which affect factors such as reactive oxygen species (ROS), diacylglycerol (DAG), tumor necrosis factor‐α (TNF‐α), and free fatty acid (FFA).^15^, ^16^

MiRNAs (microRNA) are also new key factors in regulating inflammatory response and normal functioning of the immune system.^14^ Several important obesity‐related inflammatory miRNAs are involved in a variety of obesity‐related cancers.^14^ Evidence suggests that miRNAs play important role in the pathogenesis of CRC.^17^ Recently, the effect of inflammatory miRNAs as a common factor between obesity and cancer was reviewed.^14^ The previous studies found the association of miRNAs targetome variants (targetome means: miRNA binding sites, along with their ± 25 nucleotide regions) on different types of multifactorial diseases such as CRC and obesity. The genetic variations in these regions are very important in binding miRNA on its target gene, thus these variations could change the miRNA regulatory function on the target gene.^18^

Investigating the overlap of genetic and epigenetic factors in obesity and cancer can be effective for finding their unknown associated causes and mechanisms involved in the increased incidence of cancer in obese people. In the present study, the results of several reviews were combined with bioinformatics interactions (miRNA:mRNA) to identify candidate genes and miRNAs involved in these conditions. Finally, the role of inflammatory miRNA and genes, as well as their interactions in PBMCs samples from CRC and obesity subjects was experimentally examined.

## 2 METHODS

### 2.1 Study design and study population

**Figure 1:**
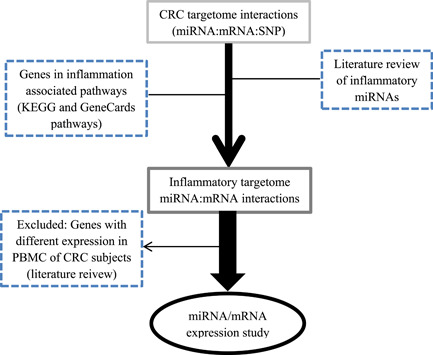
Study workflow. The figure should show the selection process for miRNAs and mRNAs that were included in the case control study. CRC, colorectal cancer; KEGG, Kyoto Encyclopedia of Genes and Genomes; miRNA, microRNA; mRNA, messeger RNA; PBMC, peripheral blood mononuclear cells; SNP, Single nucleotide polymorphism.

An overview of the workflow is shown in Figure 1. Step 1: Identifying the CRC targetome miRNA:mRNA interactions. The previously identified bioinformatics CRC targetome interactions (miRNA:mRNA:SNP) for CRC,^19^ which were selected by using genetic association studies (GWAS) data (https://www.ebi.ac.uk/gwas/), the 1000 Genome project, StarBase (version 2, available at http://starbase.sysu.edu.cn/starbase2/index.php), targetScan (version 7.1, available at http://www.targetscan.org/vert_71/), and microRNA.org (available at: http://www.microrna.org/microrna/home.do) were gathered. Briefly, the CRC‐associated variants identified by EBI‐NHGRI GWAS catalog (CRC‐associated GWAS variants). Then information from the 1000 Genome project was used to identify variants in linkage disequilibrium (LD) with CRC‐associated GWAS variants. To identify CRC targetome interactions (miRNA:mRNA), the genomic position of miRNA:mRNA interactions from the StarBase, targetScan, and microRNA.org databases were compared with the above identified variants. Step 2: Literature review of inflammation associated miRNA and genes. A list of important miRNAs involved in the regulation of the immune system and inflammation (inflammatory‐miRNAs) was prepared by review of previously published articles (literature search performed, Supplementary Method, Table S1). Also, genes showing expression changes in PBMC or blood of CRC subjects in previously published articles were investigated (literature search performed, Supplementary Method, Table S2). The genes involved in inflammation‐associated pathways were selected based on method described in the previously published article^20^ by assessing the Kyoto Encyclopedia of Genes and Genomes (KEGG) and GeneCards databases. Step 3: Identifying inflammatory CRC targetome interactions (inflammatory miRNA:mRNA). To select inflammatory CRC targetome interactions, the inflammation‐associated genes and miRNAs identified by literature review (from Step 2) were examined in the CRC targetome miRNA:mRNA interactions (from Step 1). The results showed miR‐506‐3p:*LAMC1*, miR‐124‐3P:*LAMC1*, miR‐150‐5p:*LAMC1*, miR‐506:*GNB3*, miR‐124:*GNB3*, and miR‐150:*GNB3* as candidate inflammatory CRC targetome interactions for experimental study. Accordingly, four miRNAs (miR‐506‐3p, miR‐150‐5p, miR‐124‐3p, and miR‐10b) and two messenger RNAs (mRNAs) (*LAMC1* and *GNB3*) shortlisted for expression study using real time polymerase chain reaction (PCR) quantification in CRC patients.

A case‐control association study was designed to examine these potential markers on clinical samples. The study population included 60 CRC patients and 60 normal subjects. Only subjects over 40 years old were included in the study. CRC patients were diagnosed based on pathology reports of the biopsy taken from the site of tumor after colonoscopy in the Department of Gastroenterology, Imam Khomeini Hospital. Newly diagnosed patients without previous chemotherapy and radiation therapy were included in the CRC group. The control group contained healthy individuals (free from any type of cancers or specific diseases) who were referred to the Valiasr Laboratory of Imam Khomeini Hospital for a routine checkup and had no history of CRC. Individuals with one of the following criteria were excluded from this study: the decision to leave the study, body mass index (BMI)< 18.5, less than 40 years, women with infant children, pregnant women, inflammatory bowel disease (IBD) familial adenomatous polyposis (FAP), Peutz‐jeghers syndrome (PJS), Lynch syndrome (HNPCC), MUTYH‐associated polyposis, and Turbot syndrome subjects. After signing the informed consent form, 5 ml blood was taken from each subject. This study was performed according to the ethical guidelines of medical genetic research in the Islamic Republic of Iran, the Ministry of Health and Medical Education, and was approved by the Research Ethics Committee of Endocrinology and Metabolism Research Institute, Tehran University of Medical Sciences (IR. TUMS. EMRI. REC.1396.00198).

### 2.2 PBMCs isolation

The blood was collected between 8:00 p.m. and 10 p.m. (Pre Midnight) in a 6ml EDTA K2 tube (Greiner Bio‐One) from the participants who were fasting. Isolation of peripheral blood mononuclear cells was performed 2–6 h after sampling. For this purpose, blood samples were transferred to the genetic laboratory for isolation by maintaining cold conditions. First, 3 ml blood was added to the falcon containing 3 cc ficoll (cedarlane). In this method, a blood sample was added slowly with a glass pipette, at a steady speed with an angle of 45° to the ficoll. In the next step, the sample was centrifuged at 3000 RPM for 20 min. After that, 6 cc of saline phosphate buffer was poured into a 15 ml falcon and the second phase from the previous step was added to it. The centrifuge was repeated for 20 min. The supernatant was then completely discarded and the pellet was dissolved in 0.5 cc of trizol (Invitrogen) and stored at −80°C for total RNA extraction.

### 2.3 RNA extraction

Total RNA was extracted with trizol using the standard protocol. Some changes were made to the original protocol to obtain miRNAs along with other longer RNAs. In this method, 100 μl of chloroform was added to PBMCs and the sample was vortexed for 15 s. The sample was then placed away from the light for 3 min. For the next step, the centrifuge was performed with a speed of 12,000*g* and 4°C for 15 min. The colorless phase was carefully transferred to a 1.5 ml tube and the same volume of isopropanol was added. After inverting the samples several times, it was placed at −20°C for 10min and then centrifuged at 12,000*g* at 4°C for 10 min. After draining the supernatant, one ml ethanol (75%) was added to it and was vortexed for a few seconds, afterward the centrifuge was performed for 10 min at 12,000*g* at 4°C. Finally, ethanol was discarded, diethyl pyrocarbonate (DEPC)‐treated water was added to the resulting pellet, and RNA concentration and quality were checked by NanoDrop.

### 2.4 Gene‐specific complementary DNA (cDNA)

To obtain specific sequences of target genes, cDNA synthesis was performed manually. For this purpose, reverse primers and reverse transcription primers related to the studied genes and miRNAs were designed (Supplementary Method, Table S3). The mentioned primers reached 5 pmol/μl concentrations and then 2 μl of each primer was combined with 2 μl DEPC‐treated water (mixture primer). For cDNA synthesis, 1 μl of a mixture primer with 3.75 μl of DEPC‐treated water, 0.25 μl reverse transcription enzyme M‐MLV (Yekta Tajhiz Azma), 2 μl reverse transcription enzyme buffer, 2 μl of dNTP (Yekta Tajhiz Azma), and 1 μl total RNA were mixed. The PCR was performed with the following program: 30 min 16°C, 30 min 42°C, and 5 min 85°C. Finally, the PCR product was diluted with 10 μl of distilled water and stored at −70°C.

### 2.5 Assessing the association of selected genes, and miRNAs with CRC and obesity

#### 2.5.1 Quantitative real‐time PCR

Quantitative real‐time PCR was applied to evaluate selected gene expression and the level of miRNAs. Specific primers for the target genes were designed and synthesized (Supplementary Method, Table S4). The glyceraldehyde‐3‐phosphate dehydrogenase (GAPDH) gene was used as a reference gene for mRNA expression and SNORD‐44 and SNORD‐47 were used as reference genes for the miRNA expression. The real‐time PCR reaction was performed in a volume of 10 μl, which included 5 μl of FastStart Essential DNA Green Master 2×, 0.5 μl of forwarding primer, 0.5 μl of reverse primer, 3 μl of dH2O and 1 μl of cDNA (program related to gene expression and miRNA levels is presented in Supplementary Method, Table S5). The reactions were performed with Light Cycler 96 (Roche) in two replications and the *C*_t_ value was calculated by Light Cycler 96 software. Product specificity was confirmed by melting curves and gel electrophoresis.

### 2.6 Statistical analysis

Statistical analysis related to demographic characteristics between patient and healthy groups was performed with R programming language (version 3.4.2) and IBM SPSS Statistics 22 (SPSS Inc.). Comparisons between the two groups for continuous variables and categorical variables were performed by *t*‐test and *χ* ^2^, respectively. To evaluate miRNA levels and gene expression, expression changes were first obtained by the $2^{‐\Delta \Delta C_{t}}$ method. Then, since the data were nonparametric (Kolmogorov–Smirnov test), the Mann–Whitney *U* test method was used for two‐group analysis, and the Kruskal–Wallis *H* test method was used for multigroup analysis.

## 3 RESULTS

### 3.1 Clinical characteristics of subjects included in the study

The demographic characteristics of the individuals participating in the miRNA and mRNA expression study (*N* = 120) are given in Table 1.

**Table 1:** Demographic characteristics of study participants

Characteristics	CRC (*N* = 60)	Control (*N* = 60)	*p* Value
Age (year)	60.47 ±14.27	54.63 ± 10.45	.012
25 > BMI	60.17 ± 18.90	58.53 ± 9.94	.721
25–29	62.81 ±11.14	53.32 ± 8.48	.002
30 ≤ BMI	57.67 ± 8.79	52.78 ± 12.77	.219
≥60, years (%)	55.00	30.00	.006
Sex, male (%)	51.67	41.67	.272
25 > BMI	20.00	16.67	
25–29	16.67	21.67	
30 ≤ BMI	15.00	3.33	
BMI (kg/m^2^)	26.88 ±4.59	27.49± 4.20	.447
25 > BMI	40.00	28.33	
25–29	35.00	41.67	
30 ≤ BMI	25.00	30.00	
Family history of cancer (%)	20.00	31.67	.364
25 > BMI	6.67	5.00	
25–29	8.33	15.00	
30 ≤ BMI	5.00	11.67	
Diabetes (%)	11.67	25.00	.018
25 > BMI	5.00	5.00	
25–29	5.00	13.33	
30 ≤ BMI	1.67	6.67	
Smoking (%)	5.00	16.67	.007
25 > BMI	1.67	6.67	
25–29	3.33	10.00	
30 ≤ BMI	0.00	0.00	
High blood pressure (%)	13.33	15.00	.684
25 > BMI	6.67	1.67	
25–29	3.33	5.00	
30 ≤ BMI	3.33	8.33	
High blood fats (%)	0.00	1.67	.497
25 > BMI	0.00	1.67	
25–29	0.00	0.00	
30 ≤ BMI	0.00	0.00	
History of cardiovascular disease (%)	18.33	23.33	.381
25 > BMI	8.33	5.00	
25–29	8.33	11.67	
30 ≤ BMI	1.67	6.67	
History of thyroid disease (%)	3.33	11.67	.016
25 > BMI	0.00	5.00	
25–29	1.67	5.00	
30 ≤ BMI	1.67	1.67	

*Note*: The age of the patient group is related to the time of diagnosis and the age of the control group is related to the time of participation in the study. Quantitative variables are specified as mean± standard deviation and qualitative variables are specified as a percentage. For all factors, the percentage of people in each group is also reported based on BMI classification.

Abbreviations: BMI, body mass index; CRC, colorectal cancer.

### 3.2 Identifying candidate mRNA

The literature searches (Supporting Information: Table S2) were performed to find genes that previously were associated with CRC in PBMCs or whole blood (The results are shown in Supplementary Results, Table S6).

Then, the CRC interactions in miRNA targetome (obtained by our previous bioinformatics article^19^) were integrated with the results in Supporting Information: Table S6. Finally, no miRNA binding site interactions were found for these genes. This shows that the genes which show interactions in the bioinformatics analysis have not been studied on CRC in previous studies.

To find the inflammatory genes, the functions of the genes in these interactions were assessed based on inflammation‐related pathways (as described in the method) and the results showed *LAMC1* (Laminin subunit ɣ‐1) and *GNB3* (G protein subunit β‐3) genes as candidate genes.

### 3.3 Identifying candidate miRNAs and their interactions

For this purpose, we performed literature searches (Supporting Information: Table S1) on miRNAs associated with inflammatory reactions. The results are shown in Supplementary Results, Table S7.

The interactions of candidate genes (*LAMC1* and *GNB3*) with the most important inflammatory miRNAs listed in Supporting Information: Table S7 were examined.

Finally, miR‐150‐5p, miR‐506‐3p, miR‐124‐3p based on their roles in inflammation and interactions with *LAMC1* and *GNB3* (interactions are miR‐150:*LAMC1*, miR‐150:*GNB3*, miR‐506:*LAMC1*, miR‐506:*GNB3*, miR‐124:*LAMC1*, and miR‐124:*GNB3*), besides miR‐10b based on its main role in cancers and inflammation have been selected.

### 3.4 Association of selected interactions with CRC and/or obesity

Product specificity was confirmed by the assessing primer–dimers (melting curve) and gel electrophoresis. The results showed that only the specific products were reproduced. The melting curve associated with each gene and miRNA is shown in Supplementary Results, Figures S1 and S2. The gel electrophoresis images are also presented in Supporting Information: Figures S3 and S4.

### 3.5 Association of selected genes with CRC/obesity

**Figure 2:**
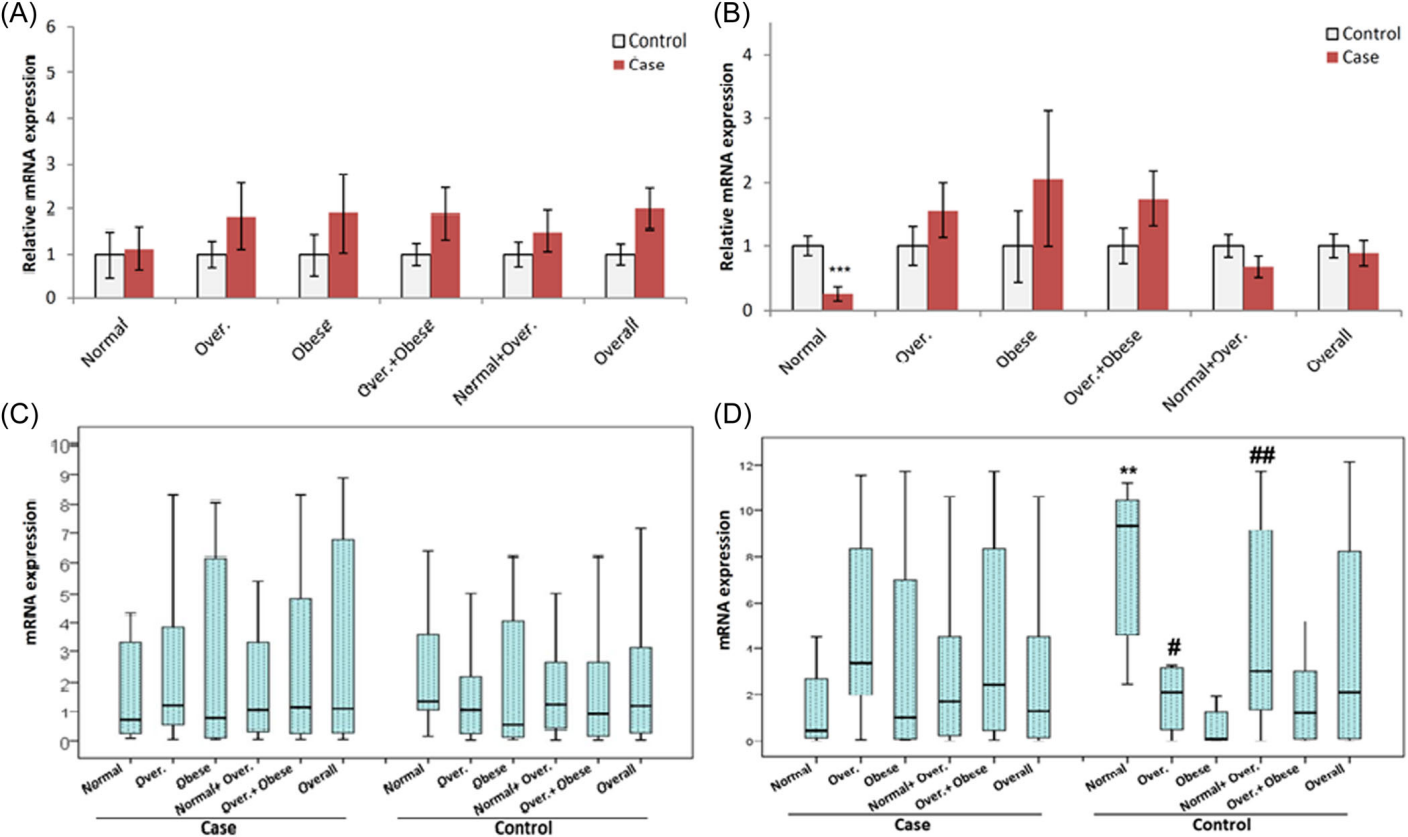
*GNB3* and *LAMC1* gene expressions: (A) and (B) are shown relative expressions between CRC and Control (CRC vs. Control) for *GNB3* and *LAMC1*, respectively. (C) and (D) are shown expression within CRC or Control BMI subgroups for *GNB3* and *LAMC1*, respectively. BMI, body mass index; CRC, colorectal cancer; *GNB3*, G protein subunit β‐3; LAMC1, laminin subunit γ‐1. **p < .01 with overweight, obese, and normal + overweight groups. ^#^ *p <*.01, ^##^ *p <*.001 with the obese group.

The *GNB3* and *LAMC1* expressions are shown in Figure 2. While the *GNB3* mRNA expression was increased in CRC subjects compared to control, the result was not statistically significant. *LAMC1* mRNA expression was associated with weight (*p <* .05). Normal‐weight subjects showed increased gene expression compared with overweight and obese subjects (*p <* .01). Also in the CRC group, the normal‐weight subjects had significantly lower LAMC1 mRNA expression compared with normal‐weight control subjects (*p <* .001).

### 3.6 Association of selected miRNAs with CRC/obesity

**Figure 3.**
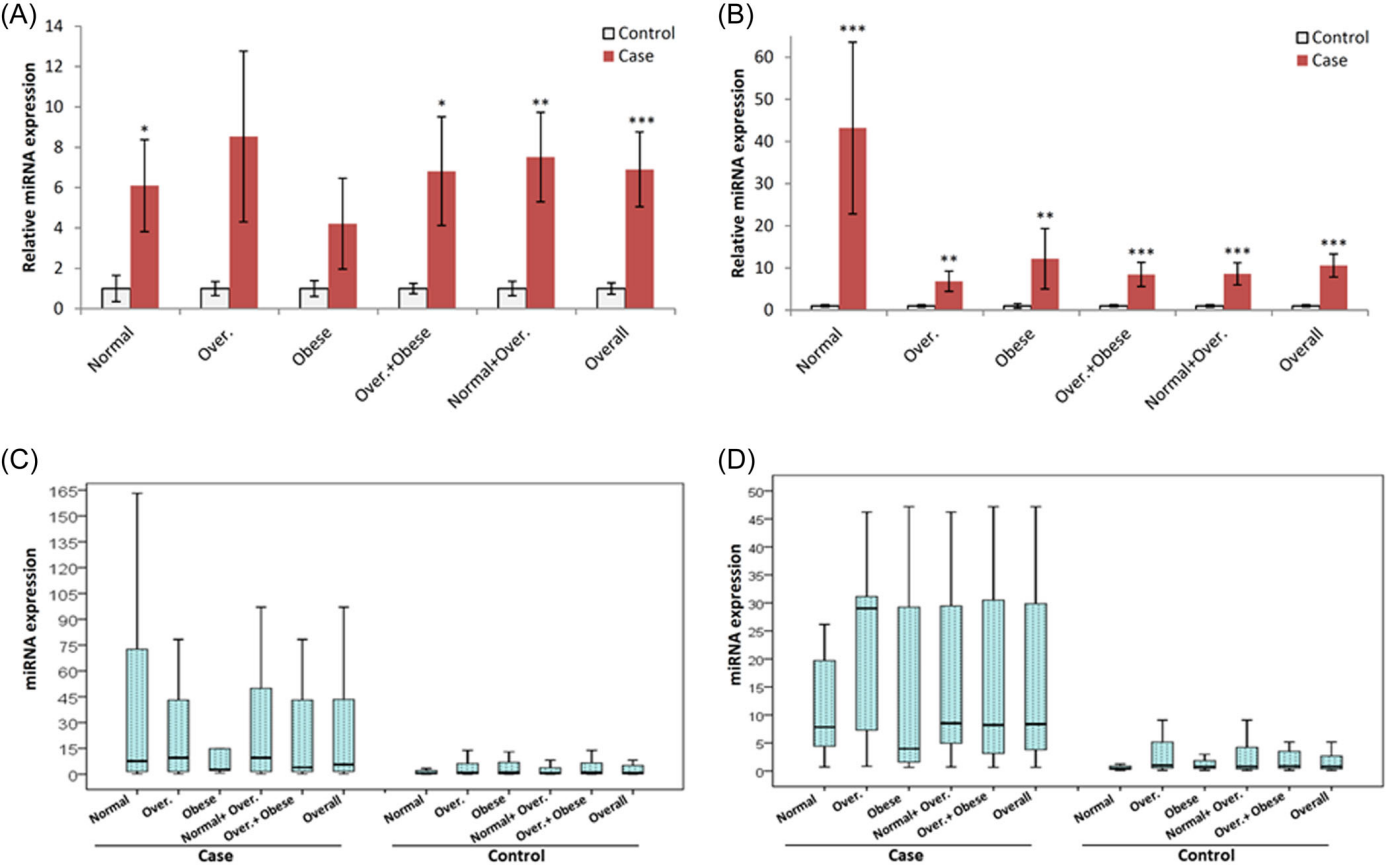
miR‐506 and miR‐124 levels (SNORD‐44 as reference gene): (A) and (B) compare relative level between CRC and Control (CRC vs. Control) for miR‐506 and miR‐124, respectively. (C) and (D) compare CRC or Control BMI subgroups for miR‐506 and miR‐124, respectively. BMI, body mass index; CRC, colorectal cancer; miRNA, microRNA. *p <.05, **p < .01, ***p < .001 with control.

**Figure 4.**
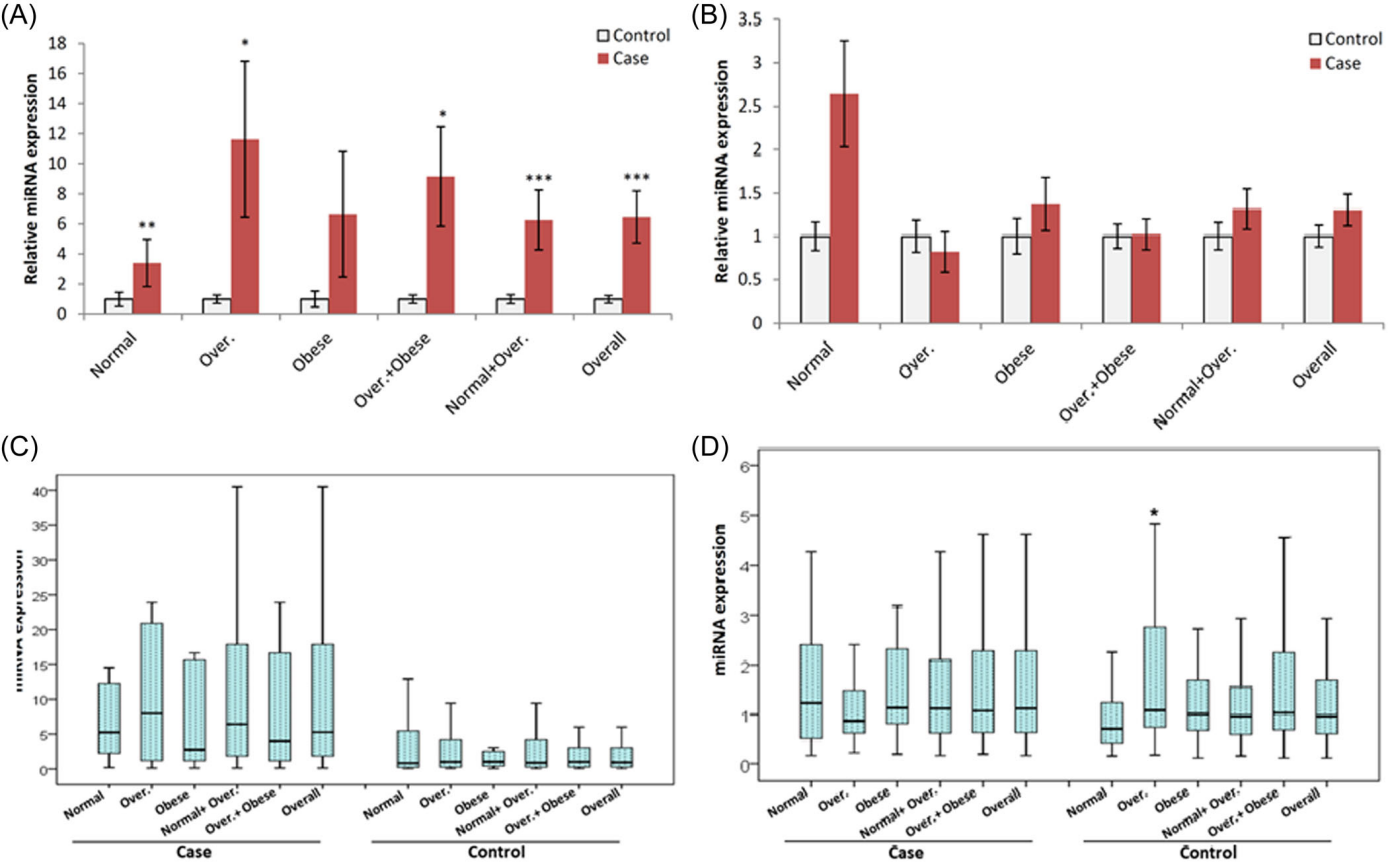
miR‐10b and miR‐150‐5p levels (SNORD‐44 as reference gene): (A) and (B) compare relative level between CRC and Control (CRC vs. Control) for miR‐506 and miR‐124, respectively. (C) and (D) compare CRC or Control BMI subgroups for miR‐506 and miR‐124, respectively. BMI, body mass index; CRC, colorectal cancer; miRNA, microRNA. *p < .05, **p < .01, ***p <.001 with control group.

The expression of miR‐506 significantly increased in the CRC group compared to the control (*p <* .001). There was no significant association between CRC or control subjects stratified in terms of BMI. The results are shown in Figure 3 and Supporting Information: Figure S5. The expression of miR‐124 significantly increased in the CRC group compared to control in different BMI groups (*p <* .001). However, there was no significant association between CRC or control subjects stratified in terms of BMI. The results are shown in Figure 3 and Supporting Information: Figure S6. The expression of miR‐10b significantly increased in the CRC group compared to the control (*p <*.001). The results are shown in Figure 4 and Supporting Information: Figure S7. There was no significant association between CRC and control groups for the expression of miR‐150. However, normal subjects had lower miR‐150 levels than overweight subjects (*p <* .05) in the control groups. The results are shown in Figure 4 and Supporting Information: Figure S8.

As there was a significant difference between the numbers of subjects in ages less than 60 years (40–59) with ages more than 60 (≥60), we performed another analysis to find the possible effect of age on our results. The results are shown in, Table 2. The results for miR‐506 seems to be associated with age, the results for subjects less than 60 years was not significant in SNORD‐44 reference groups (*p* < .05), and showed weak significant results for SNORD‐47 groups (*p* < .05), while the results for more than (*p*≤ .001).

**Table 2:** miRNAs and genes expression results according to the age classification

	40–59, years		Age ≥ 60, years	
miRNAs/genes	SNORD‐44	SNORD‐47	SNORD‐44	SNORD‐47
miR‐10b	**0.007**	**0.019**	**0.001**	**0.001**
miR‐124	**0.000**	**0.001**	**0.000**	**0.000**
miR‐150	0.156	0.209	0.827	0.108
miR‐506	0.052	**0.049**	**0.001**	**0.001**

	GAPDH		GAPDH	
*GNB3*	0.538		0.430	
*LAMC1*	0.903		0.540	

*Note*: Bold *p* values are statistically significant (*p* < 0.05).

Abbreviations: *GNB3*, G protein subunit β‐3; *LAMC1*, laminin subunit γ‐1; miRNA, microRNA.

## 4 DISCUSSION

In this study candidate genes and miRNAs were selected based on miRNA:mRNA interactions using bioinformatics, literature searches, and pathway analysis. Inflammation was used as a common pathogenic mechanism between obesity and CRC to finally select the genes and miRNAs involved in both conditions. *LAMC1* and *GNB3* genes and four miR‐150‐5P, miR‐506, miR‐124, and miR‐10b were identified based on in silico and literature search approaches.

The results of the present study showed that the expression of the *LAMC1* gene is reduced in the normal weight CRC group. The expression of this gene in the control group was also affected by weight and was decreased in the obese group. In previous studies, the relationship between polymorphisms of *LAMC1* or *GNB3* and CRC have been investigated.^20^, ^21^, ^22^ The expression of this gene was examined in tissue samples of other cancers such as cervical squamous cell carcinoma, hepatocellular carcinoma.^23^ In CRC cell lines in the groups with increased miR‐506 levels, a decrease in the level of the *LAMC1* gene was observed.^24^ In the present study, the same result was also observed in the normal weight CRC group. Overall, there was no specific study on the expression of this gene in PBMCs samples of CRC patients and the present study suggests that expression of this gene changes only in normal‐weight CRC subjects.

We observed that the level of miR‐150‐5p was significantly increased in overweight and obese subjects, which is in line with previous studies. It has been shown that miR‐150 levels increase in subcutaneous adipose tissue of obese individuals.^25^ This miRNA plays an essential role in regulating B cells function in adipose tissue, which ultimately regulates both metabolic and immune‐dependent homeostasis in adipose tissue.^26^ In this regard, it was shown that the level of miR‐150 was increased in the colon tumors of obese mice.^27^ However, in a rare case, one study showed that the level of free miR‐150 in the blood was decreased in obese and overweight groups compared to the control group.^28^ While the present study did not show significant relation for miRNA‐150‐5p expression in CRC, previous studies have shown different results. Studies on CRC cell lines have shown that decreased expression of this miRNA increases epithelial‐mesenchymal transition  cell invasion.^29^ Other studies have shown that miR‐150‐504‐519d induces invasion and induction of cell death in the SW48 cell line,^30^ and reduces the invasion of LoVo and HCT‐116 cell lines.^31^ This miRNA also inhibits tumor progression in CRC tissue.^32^ However, it should be noted that none of the mentioned studies examined the level of miR‐150‐5p in PBMCs.

Previous studies mostly have shown that miR‐10b‐5p is effective in the progression, metastasis, and invasion of various cancers and has a carcinogenic function, its expression is also increased in CRC.^33^ In some studies, its expression is dependent on the tumor node metastasis stage, and in cases with liver metastasis, its expression is much higher in the CRC cell line, so that the increase in its expression predicts clinical‐pathology features, and is suggested as a noninvasive marker in the prognosis, metastasis and of course the therapeutic goal of CRC.^34^, ^35^ Inhibition of this miRNA prevents liver metastasis, tumor growth and reduces cyclone D1, which induces cell cycle arrest and apoptosis. This miRNA is effective in increasing the invasion and migration of CRC tumor cells through various targets.^36^ A meta‐analysis also showed that increased expression of this miRNA indicates a decrease in overall survival in patients with various types of cancer, including CRC.^37^ The level of this miRNA in liver metastasis was decreased compared to primary cancer^38^ and in another study, its expression was decreased in CRC cell lines, in a way that increases in its expression suppressed metastasis, colony formation, and cancer cell migration.^39^ In general, previous studies confirm the results of the present study. There are no adequate studies on the level of miR‐10b‐5p in obesity, but some studies have shown that its level in the cancerous tissue of obese people with breast cancer is lower than normal cancer patients^40^ and others showed a decrease in its level in obese conditions.^41^

The results of the present study showed that the levels of miR‐506‐3p, miR‐124‐3p, and miR‐10b‐5p were increased in the CRC groups, while they had no significant association with obesity. As our results showed the increased level of miR‐506‐3p is associated with age and only observed in ages more than 60 years groups.

Previous studies showed that the level of miR‐506 increases significantly in both tumor tissue and plasma samples in patients with colon cancer. It could be effective in the occurrence and progression of cancer and can be considered as a possible marker.^42^ In studies performed on blood samples obtained from CRC patients without chemotherapy or radiation therapy, a significant increase in the level of miR‐506 was observed compared to healthy individuals, and the level of this miRNA was proposed as a marker in the initial diagnosis of CRC.^43^ However, the level of this miRNA in tumor tissue was decreased compared to normal tissue in another study.^44^ Given the controversial results in previous studies, the present study on PBMCs samples confirmed the increase in miR‐506 levels in CRC patients.

According to our knowledge, no studies have been done on the role of miR‐124‐3p in obesity. The results of previous studies on CRC patients are contrary to the results of the present study. In previous study, the level of this miRNA in tumor tissue was decreased compared to normal tissue,^45^ and in vitro increase of its level resulted in more CRC cell death compared with controls^46^ and inhibition of tumor progression.^44^

## 5 CONCLUSION

In the present study, the expression of miR‐10b‐5p, miR‐506, and miR‐124 were increased in PBMCs of CRC patients in the Iranian population, which may have a regulatory effect on the reduction of *LAMC1* expression and thus affect CRC. It seems that the expression of miR‐150‐5p is associated with BMI. In miR‐506‐3p:*LAMC1* and miR‐124‐3P:*LAMC1* interactions, *LAMC1* expressions, miR‐506 and miR‐124 levels were significantly associated with CRC. It is suggested that changes in the levels of these miRNAs may affect the expression of *LAMC1* in CRC patients. However, in the case of miR‐150:*GNB3*, miR‐506:*GNB3*, and miR‐124:*GNB3*, only miR‐124 and miR‐506 were associated with CRC, suggesting that this interaction may not be effective in CRC and obesity. These results for the first time showed the role of inflammatory miRNA:mRNA interactions on the risk of CRC or/and obesity. However, the mechanism of these interactions and role of these genes and miRNAs should be investigated in future functional studies. This study only assessed these associations in Iranian subjects, further studies are recommended to investigated these interactions on other populations and larger samples.

## AUTHOR CONTRIBUTIONS

All authors contributed to the study's conception and design. Reza Taslimi, Zeinab Rahmani, Alireza Kazemeini, and Roobic Behboo diagnosed the diseases, provided blood samples and clinical data. Morteza Gholami, Marziyeh Zoughi, Shirin Hasani‐Ranjbar, Farideh Razi and Mahsa M. Amoli carried out the experiment. The data collection and analysis were performed by Morteza Gholami, Bagher Larijani, Milad Bastami, and Rasoul Abdollahzadeh. The first draft of the manuscript was written by Morteza Gholami, Marziyeh Zoughi and Farideh Razi with support from Mahsa M. Amoli and all authors completed and commented on previous versions of the manuscript and discussed the results. Mahsa M. Amoli supervised the project and approved the final version. All authors collaborated in revise of the manuscript and approved the final manuscript.

## CONFLICT OF INTEREST

The authors declare no conflict of interest.

## ETHICS STATEMENT

This study was performed according to the ethical guidelines of medical genetic research in the Islamic Republic of Iran, the Ministry of Health and Medical Education, and was approved by the Research Ethics Committee of Endocrinology and Metabolism Research Institute, Tehran University of Medical Sciences (IR.TUMS.EMRI.REC.1396.00198).

## Supporting information

Supplementary information.

Supplementary information.

## Data Availability

The data that supports the findings of this study are available in the supplementary material of this article.
