# Supplementary material for: The role of inflammatory miRNA–mRNA interactions in PBMCs of colorectal cancer and obesity patients
Source: Immun Inflamm Dis. 2022 Oct 27;10(11):e702. doi: 10.1002/iid3.702 (PMC9609448; doi:10.1002/iid3.702)
Supplement: Supplementary file 1 — Supplementary information. [file IID3-10-e702-s002.docx]

**Table S1. Search strategy: the study of inflammatory miRNAs with changed level in blood samples**

| **Search 1: PubMed^**  ((MicroRNA*[TI] OR “micro RNA”[TI] OR ((RNA[TI] OR RNAs[TI]) AND Micro[TI]) OR miRNA[TI] OR miRNAs[TI] OR miR[TI] OR miRs[ti] OR MIRN[TI] OR MIRNS[TI] OR “hsamiR” [TI] OR hsa-miR[TI] OR let[TI] OR lin[TI] OR stRNA[TI]) AND (Inflammation[MeSH] OR inflammation*[tiab] OR inflammatory[TIAB] OR (Immune and (response OR System)) OR “T cell”[tiab] OR “B cell”[tiab] OR Bcell[tiab] OR Tcell[tiab]) AND ("peripheral blood mononuclear cell*"[TIAB] OR PBMC*[TIAB] OR (BLOOD[TIAB] AND ("peripheral blood mononuclear cell*" OR PBMC* OR lymphocyte OR "T cell" OR "B cell" OR "killer cell*" OR "NK cell*" OR leukocyte OR WBC OR "white blood cell*" OR Monocyte)))) |
| --- |
| **Search 2: PubMed^**  ((MicroRNA*[TI] OR “micro RNA”[TI] OR ((RNA[TI] OR RNAs[TI]) AND Micro[TI]) OR miRNA[TI] OR miRNAs[TI] OR miR[TI] OR miRs[ti] OR MIRN[TI] OR MIRNS[TI] OR “hsamiR” [TI] OR hsa-miR[TI] OR let[TI] OR lin[TI] OR stRNA[TI]) AND (Inflammation[MeSH] OR inflammation*[tiab] OR inflammatory[TIAB] OR (Immune and (response OR System)) OR “immune system”[MeSH] OR “T cell”[tiab] OR “B cell”[tiab] OR Bcell[tiab] OR Tcell[tiab]) AND ("peripheral blood mononuclear cell*"[TIAB] OR PBMC*[TIAB] OR (BLOOD[TIAB] AND ("peripheral blood mononuclear cell*" OR PBMC* OR lymphocyte OR "T cell" OR "B cell" OR "killer cell*" OR "NK cell*" OR leukocyte OR WBC OR "white blood cell*" OR Monocyte))) AND (REVIEW[TIAB] OR SYSTEMATIC[TI] OR META[TI])) |
| **Search 3:** **Google scholar#**  PBMC + miRNA +inflammation/immune |

^ All articles (from Search 1 and 2) were scrutinized for the relevant documents by checking the title and/or abstract. After the full text of articles about the study of miRNA level in blood samples were investigated. # First 20 pages of the Google scholar search results were investigated.

**Table S2. Search strategy: the study of genes with changed expression in PMBC/blood of CRC subjects**

| **Search 1:PubMed^**  ((CRC[ti] OR colorectal [ti] OR “large intestine tumor”[ti] OR HNPCC[ti] OR ((bowel*[ti] OR intestin*[ti] OR colorect*[ti] OR rectum[ti] OR rectal[ti] OR colon*[ti] OR color*[ti] OR sigmoid*[ti]) AND (cancer*[ti] OR neoplas*[ti] OR tumor*[ti] OR tumour*[ti] OR mass[tiab] OR carcinom*[tiab] OR sarcom*[tiab] OR adenocarcinom*[ti] OR adenomatosum[ti] OR adenom*[ti] OR malignan*[ti] OR ONCOL*[ti] OR carcinogenesis[ti]))) AND (("peripheral blood mononuclear cell*"[TIAB] OR PBMC*[TIAB] OR BLOOD[TIAB]) AND ((GENE[TIAB] OR GENES[TIAB]) AND (EXPRESSION*[TIAB] OR LEVEL[TIAB])))) |
| --- |
| **Search 2:PubMed^**  ((CRC[ti] OR colorectal [ti] OR “large intestine tumor”[ti] OR HNPCC[ti] OR ((bowel*[ti] OR intestin*[ti] OR colorect*[ti] OR rectum[ti] OR rectal[ti] OR colon*[ti] OR color*[ti] OR sigmoid*[ti]) AND (cancer*[ti] OR neoplas*[ti] OR tumor*[ti] OR tumour*[ti] OR mass[tiab] OR carcinom*[tiab] OR sarcom*[tiab] OR adenocarcinom*[ti] OR adenomatosum[ti] OR adenom*[ti] OR malignan*[ti] OR ONCOL*[ti] OR carcinogenesis[ti]))) AND (("peripheral blood mononuclear cell*"[TIAB] OR PBMC*[TIAB] OR (BLOOD[TIAB] AND ("peripheral blood mononuclear cell*" OR PBMC* OR lymphocyte OR "T cell" OR "B cell" OR "killer cell*" OR "NK cell*" OR leukocyte OR WBC OR "white blood cell*" OR Monocyte))) AND ((GENE[TIAB] OR GENES[TIAB]) AND (EXPRESSION*[TIAB] OR LEVEL[TIAB])))) |
| **Search 3:PubMed^**  ((CRC[ti] OR colorectal [ti] OR “large intestine tumor”[ti] OR HNPCC[ti] OR ((bowel*[ti] OR intestin*[ti] OR colorect*[ti] OR rectum[ti] OR rectal[ti] OR colon*[ti] OR color*[ti] OR sigmoid*[ti]) AND (cancer*[ti] OR neoplas*[ti] OR tumor*[ti] OR tumour*[ti] OR mass[tiab] OR carcinom*[tiab] OR sarcom*[tiab] OR adenocarcinom*[ti] OR adenomatosum[ti] OR adenom*[ti] OR malignan*[ti] OR ONCOL*[ti] OR carcinogenesis[ti]))) AND (("peripheral blood mononuclear cell*"[TIAB] OR PBMC*[TIAB] OR BLOOD[TIAB] )AND ((GENE[TIAB] OR GENES[TIAB]) AND (EXPRESSION*[TIAB] OR LEVEL[TIAB]))) AND  (REVIEW[TIAB] OR SYSTEMATIC[TI] OR META[TI])) |
| **Search 4: Google scholar#**  PBMC + colorectal + gene + expression |

^ All articles (from Search 1, 2 and 3) were scrutinized for the relevant documents by checking the title and/or abstract. After the full text of articles about the gene expression on blood samples of CRC subjects were investigated. # First 20 pages of the Google scholar search results were investigated.

**Table S3. cDNA synthesis primers**

| **Sequence** | **miRNA/mRNA** |
| --- | --- |
| GTCGTATCCAGTGCAGGGTCCGAGGTATTCG  CACTGGATACGACTTGGCA | RT-miR-124-3p |
| GTCGTATCCAGTGCAGGGTCCGAGGTATTCG  CACTGGATACGACCACTGGT | RT-miR-150-5p |
| GTCGTATCCAGTGCAGGGTCCGAGGTATTCG  CACTGGATACGACTCTAC | RT-miR-506-3p |
| GTCGTATCCAGTGCAGGGTCCGAGGTATTCG  CACTGGATACGACCACAA | RT-miR-10b-5p |
| GTCGTATCCAGTGCAGGGTCCGAGGTATTCG  CACTGGATACGACAGTCAGTT | RT-SNORD-44 |
| GTCGTATCCAGTGCAGGGTCCGAGGTATTCG  CACTGGATACGACAACCTCA | RT-SNORD-47 |
| TCACAGGGCAGGCATTCAC | R-LAMC1 |
| GATGGCGTGCACCTTGTTG | R-GNB3 |
| TCCACCACCCTGTTGCTGTAG | R-GAPDH |

**Table S4. Genes and miRNAs primers**

| **Sequence** | mRNA/miRNA |
| --- | --- |
| F-ACACCCACTCCTCCACCTTTG  R-TCCACCACCCTGTTGCTGTAG | GAPDH |
| F-GAATGCTGACTGAACATGAAGGTCT  R-GTGCAGGGTCCGAGGT | SNORD-44 |
| F- GCGATATCACTGTAAAACCGTTCCA  R-GTGCAGGGTCCGAGGT | SNORD-47 |
| F- AGGTGGCAGATGTAAATGTAATGG  R- TCACAGGGCAGGCATTCAC | LAMC1 |
| F- GGCCACTGATTCTAAGCTGC  R- GATGGCGTGCACCTTGTTG | GNB3 |
| F- GCGTAAGGCACCCTTCTGA  R-GTGCAGGGTCCGAGGT | has-miR-506-3p |
| F- CGCATACCCTGTAGAACCGAAT  R-GTGCAGGGTCCGAGGT | has- miR- 10b-5p |
| F- ACTAAGGCACGCGGTGAAT  R-GTGCAGGGTCCGAGGT | has- miR- 124-3p |
| F- GCGTCTCCCAACCCTTGT  R-GTGCAGGGTCCGAGGT | has- miR- 150-5p |

**Table S5. Quantitative PCR program**

| Acquisition | Cycle | **Step** | Steps |
| --- | --- | --- | --- |
| - | 1 | 95°C 10 min | Pre-incubation |
| - | 40 | 95°C 10 Sec  60°C 20 Sec | Two steps amplification |
| Once | 1 | 95°C 10 Sec  65°C 60 Sec  95°C 1 Sec | Melting |
