# Supplementary material for: The role of inflammatory miRNA–mRNA interactions in PBMCs of colorectal cancer and obesity patients
Source: Immun Inflamm Dis. 2022 Oct 27;10(11):e702. doi: 10.1002/iid3.702 (PMC9609448; doi:10.1002/iid3.702)
Supplement: Supplementary file 2 — Supplementary information. [file IID3-10-e702-s001.docx]

**Table S6. The genes with changed expression in CRC patients**

| **Genes** | **Source** |
| --- | --- |
| IFNG, FOXP3, CD276, PDCD1LG2, PDCD1, CD28, ICOS, BTLA, CTLA4, LAG3, HAVCR2, PPARG, CTSL, GK, CDA, SET, PFDN5, PECAM1, APOBEC3A, UBXN11, MSL1, MMP9, BANK1, C9orf78, VPS18, ISCU, DYNC1LI2, DYM, PIP4K2B, TUG1, EPHX2, ITIH4, CDCA4, S100A8, MSH2, MSH6, PMS1, PMS2, IL10, TGFB1, IL23A, NPRL2, MDM2, DUSP6, CPEB4, MMD, EIF2S3, BCL3, IL1B, PTGES, PTGS2, MMP11, CCR1, EGR1, CACNB4, CES1, IL8, CXCL11, MAPK6, CD63 | PBMC |
|  |  |
| IL-6, ITPRIPL2, ANXA3, MS4A1, TPM4, SESTD1, TTYH3, TIMP1, CD44, TM9SF4, PIM3, PELO, SFXN3, MYL9, VCAN, TGFBI, PLXND1, TKT, PF4, IFITM3, S100A11, G6PD, AP1M1, ZC3H12A, FSCN1, NDE1, IER3, PEA15, PTP4A3, IMPDH1, PRKCDBP, ESAM, CCDC85B, IFITM2, IFITM1, COL18A1, RAB31, FLNA, TMEM158, CTSK, ENC1, ICAM1, INTS1, PI3, SLC26A2, UGDH, RANBP2, DNAJC10, SLC39A6, DIS3, ELK3, IL8, SACS, POT1, GALNACT-2, HS2ST1, XPOT, MTMR11, ETHE1, SULT1A3, SULT1A2, FCGRT, TRPM6, SULT1A1, ACADVL, COX-2, CD36, DHRS13, DUSP2, FAM198B, FKBP5, GZMB, IL1B, ITGAM, MYBL1, NEAT1, NUDT16, P2RY10, PDE4D, PDZK1IP1, SH2D2A, VSIG10, CLEC4D, IL2RB, LMNB1, PRRG4, TNFAIP6, VNN1, BANK1, CDA, TSPAN8, EPCAM, COL1A2, CDH1, LGALS4, CEACAM6, ASAP1, CDK12, CEMIP, CHCHD10, COLGALT2, FAM129C, GLIPR2, IPO5, NFKB1, PHYKPL, PXDC1, TNF, RILPL2, FRMD3, TERT | whole blood or white blood cells |

**Table S7. Inflammatory miRNAs whose levels were evaluated in blood sample studies**

| miR-9, miR-10, miR-17-3p, miR-21, miR-23a, miR-23b, miR-24, miR-27a, miR-29, miR-31, miR-34a, miR-92a, miR-106a, miR-110, miR-122, miR-124, miR-125b, miR-125a, miR-125a-5p, miR-126, miR-126-5p, miR-132, miR-133a, miR-142-3p, miR-145, miR-146, miR-146a, miR-146a/b, miR-146b, miR-147, miR-148a, miR-149, miR-150, miR-155, miR-181a, miR-181b, miR-181c, miR-181d, miR-186, miR-203, miR-210, miR-221, miR-223, miR-506, miR-551b-5p, miR-652, miR-17-92, miR-7-1, miR-9-1, miR-let-7, miR-let-7a/b/c/d/e/f/g/i |
| --- |


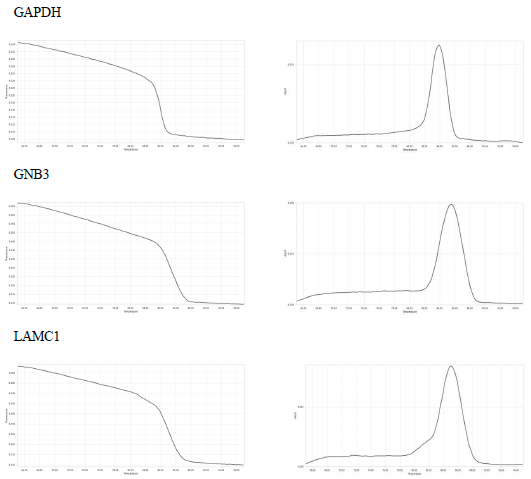


Fig. S1. Melting peak and melting carve associated to gene expression

**
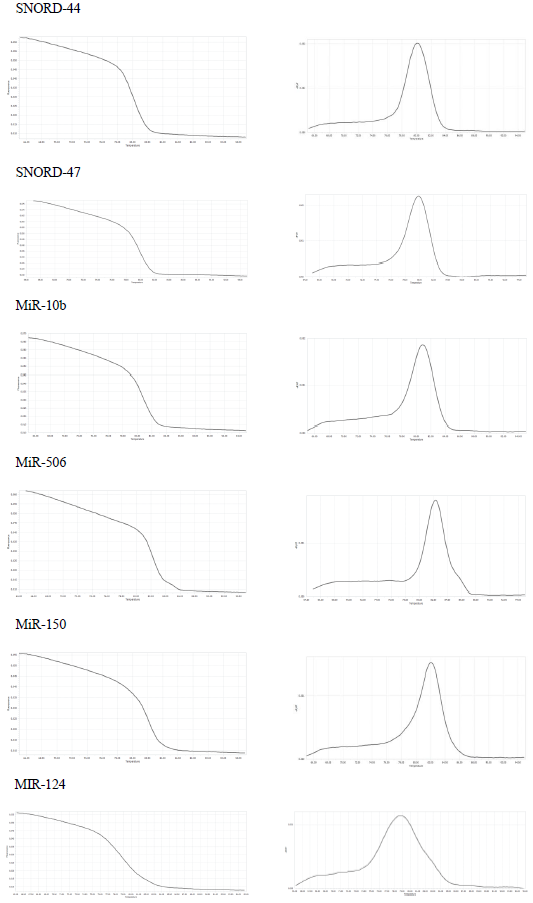
**

Fig. S2. Melting peak and melting carve associated to gene expression and miRNA level assessments

**
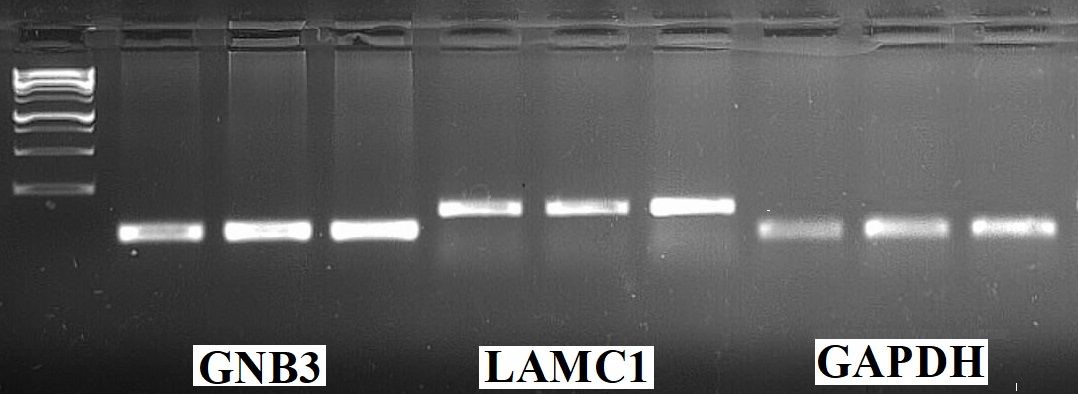
**

Fig. S3. Gel electrophoresis associated to gene expression

**
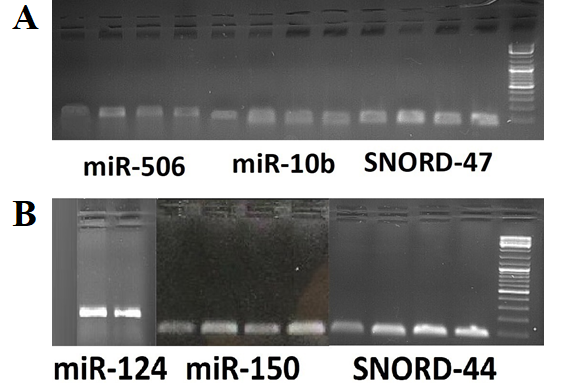
**

**B**
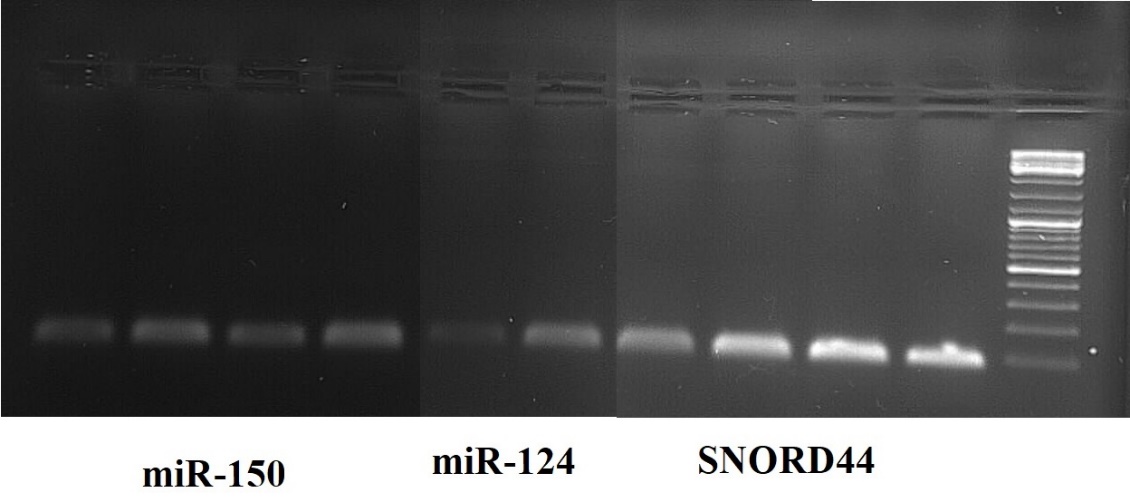


Fig. S4. Gel electrophoresis associated to miRNA level

**
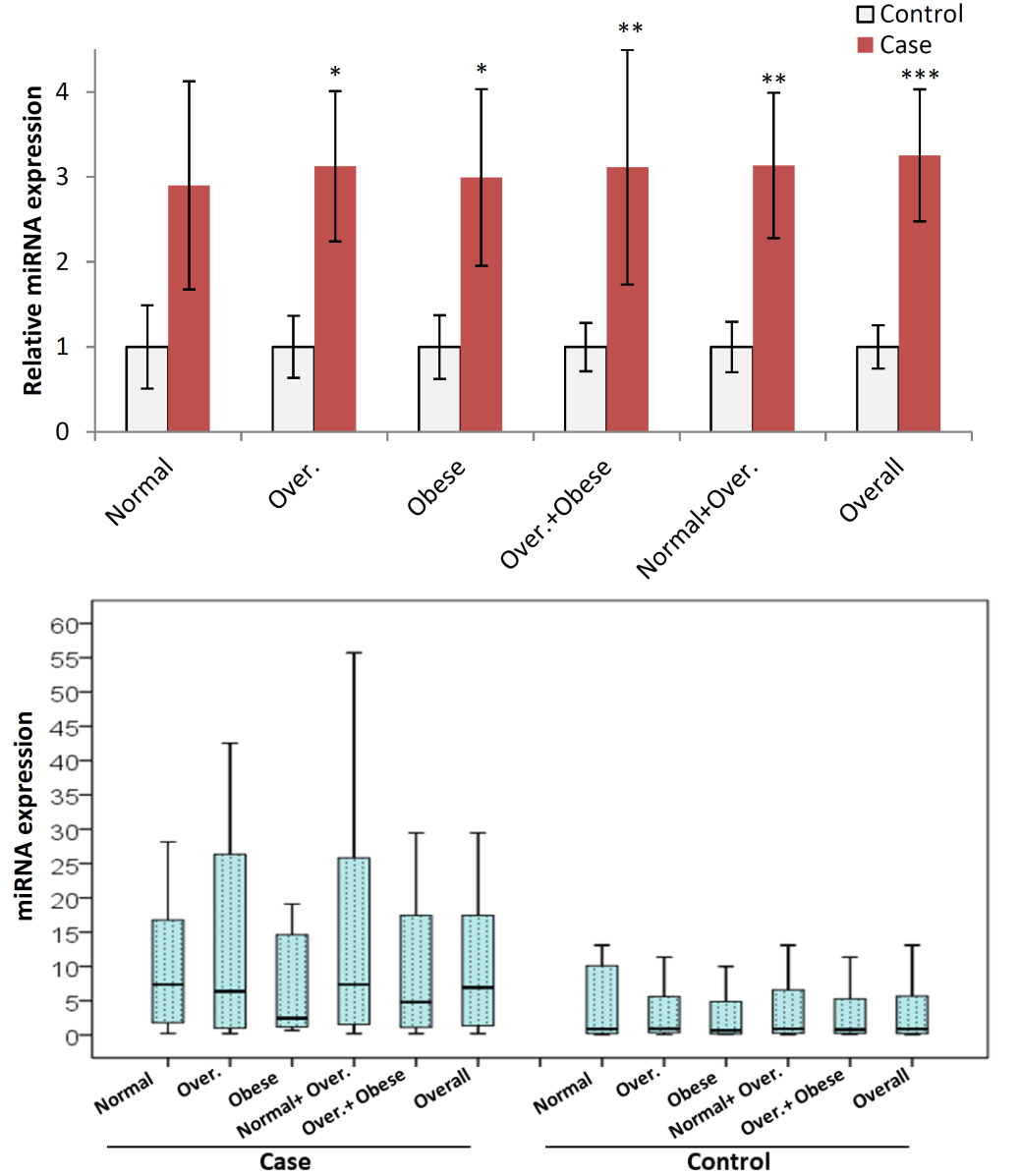
**

Fig. S5. miR-506 level compared to SNORD-47 as reference gene: A) Relative between CRC and Control (CRC vs. Control) B) within CRC or Control BMI subgroups. *p˂0.05, **p˂0.01, ***p˂0.001 with control group

**
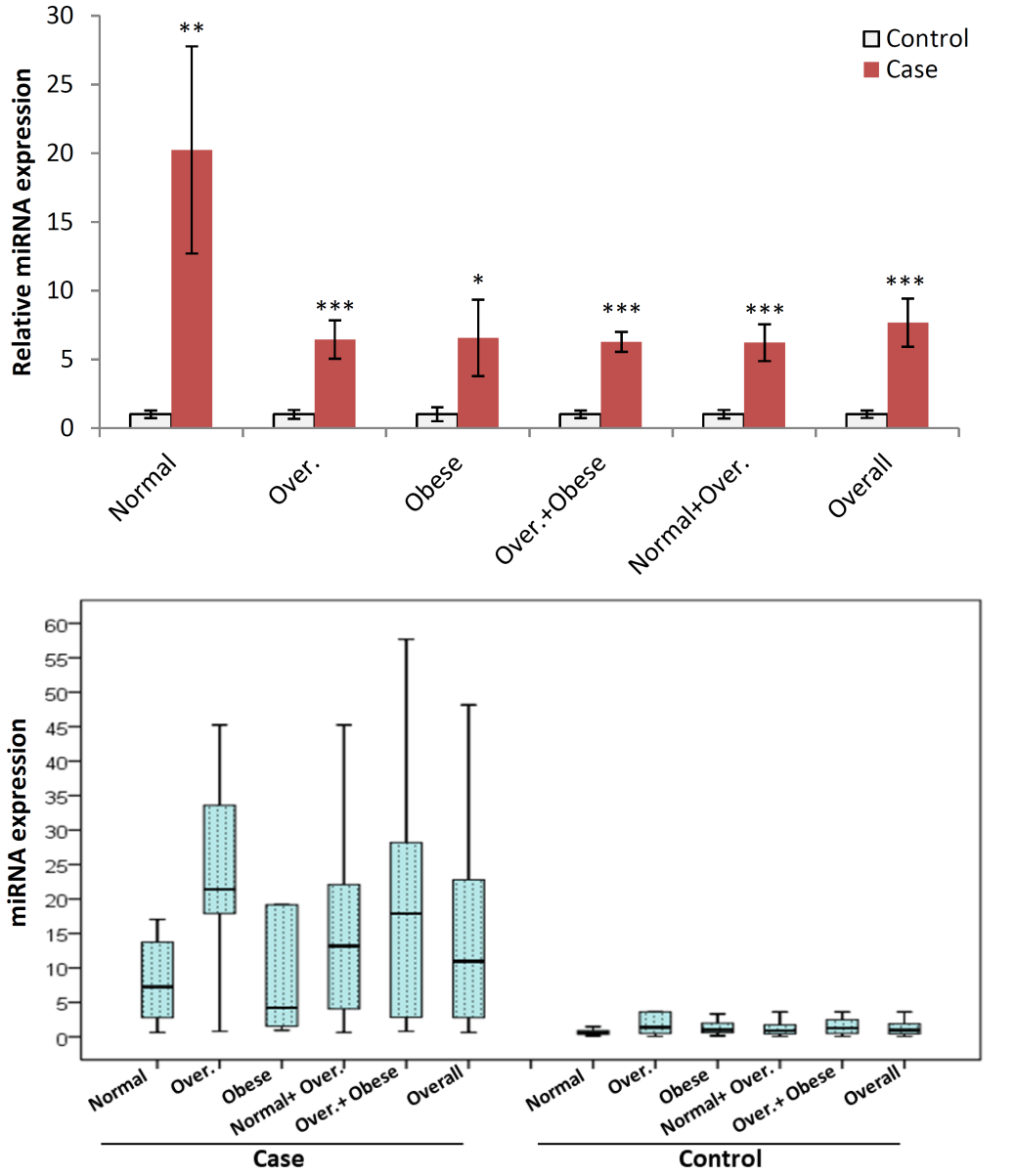
**

Fig. S6. miR-124 level compared to SNORD-47 as reference gene: A) Relative between CRC and Control (CRC vs. Control) B) within CRC or Control BMI subgroups. *p˂0.05, **p˂0.01, ***p˂0.001 with control group

**
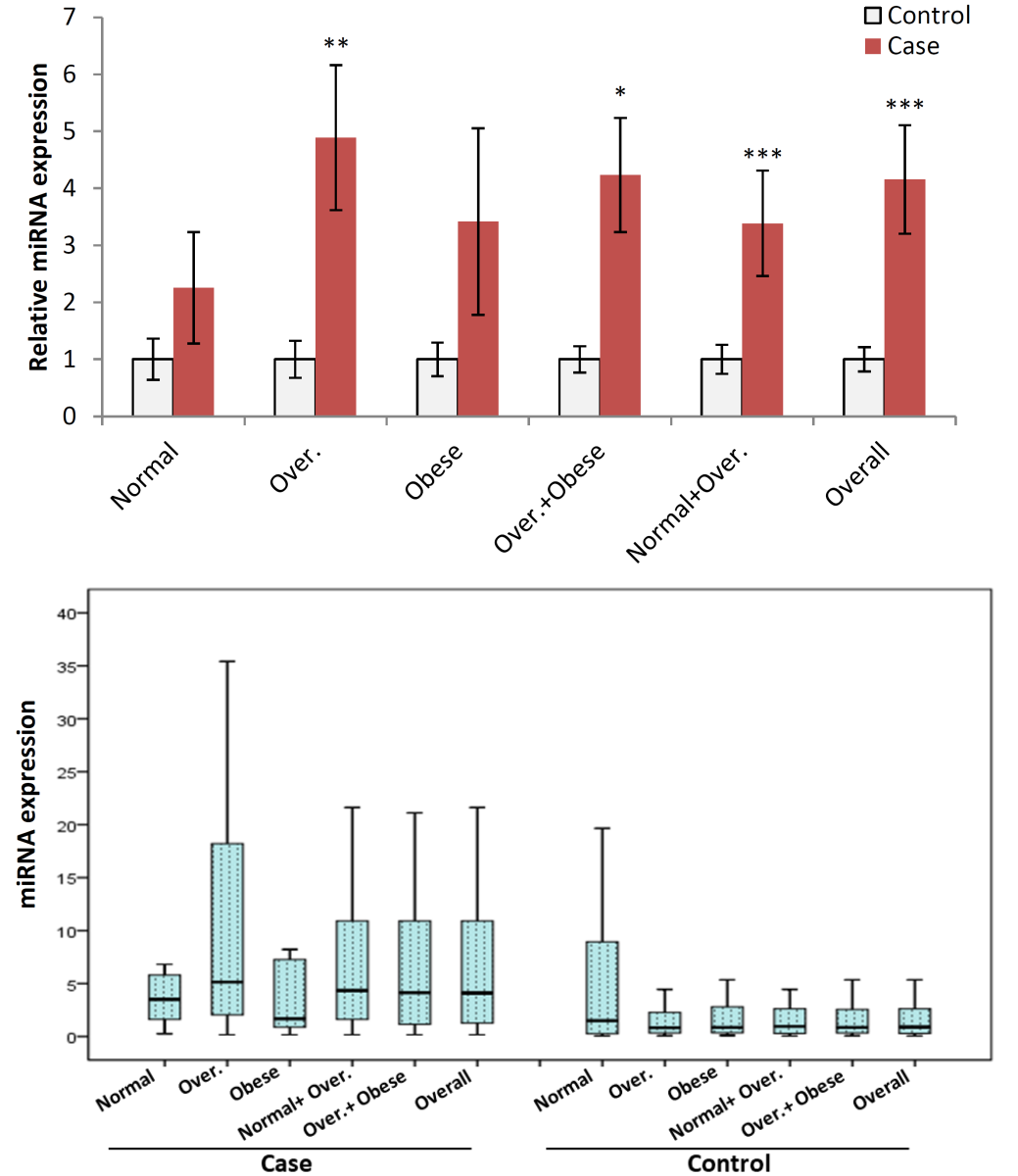
**

Fig. S7. miR-10b level compared to SNORD-47 as reference gene: A) Relative between CRC and Control (CRC vs. Control) B) within CRC or Control BMI subgroups. *p˂0.05, **p˂0.01, ***p˂0.001 with control group


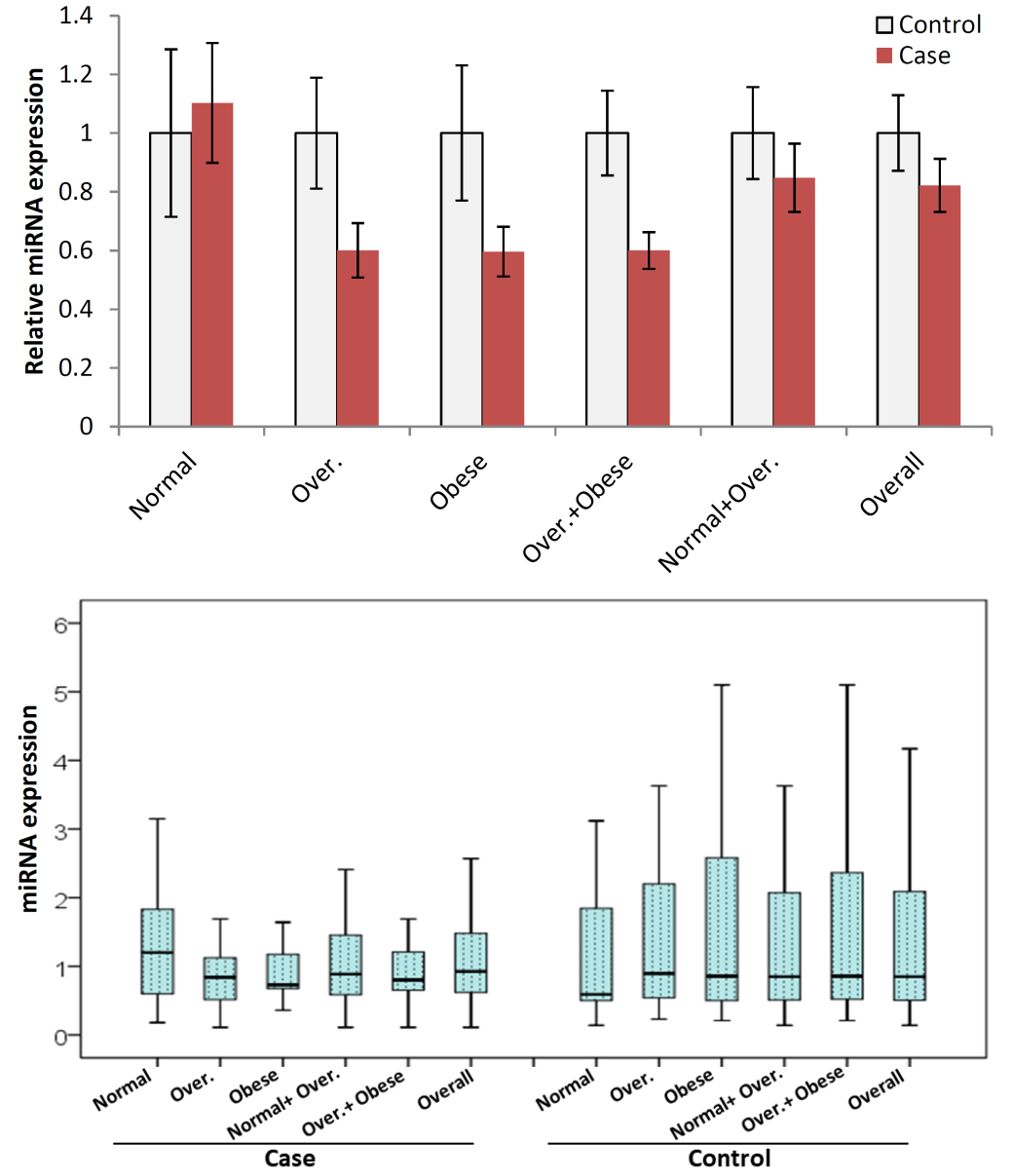


Fig. S8. miR-150 level compared to SNORD-47 as reference gene: A) Relative between CRC and Control (CRC vs. Control) B) within CRC or Control BMI subgroups.
